# Factors Influence on Pap Test Screening among Lahu Hill Tribe Women in Remote Area Thailand

**DOI:** 10.31557/APJCP.2021.22.7.2243

**Published:** 2021-07

**Authors:** Sophaphan Intahphuak, Kowit Nambunmee, Patita Kuipiaphum

**Affiliations:** 1 *Lecturer at School of Nursing, Mae Fah Luang University, Chiang Rai, Thailand. *; 2 *Lecturer at School of Health Science, Mae Fah Luang University, Chiang Rai, Thailand. *; 3 *Head of Urban Safety Innovation Research Group (USIR), Mae Fah Luang University, Chiang Rai, Thailand. *

**Keywords:** Cervical cancer, Lahu Hill tribe women, Pap test, Thailand

## Abstract

**Background:**

Papanicolaou test is widely used to screening cervical cancer but low attend rate. There is one mountainous area found high participation rate.

**Objective:**

This study aimed to determine the factors associated with successful Pap test among Lahu hill tribe women.

**Materials and Methods:**

The quantitative cross-sectional study was used in this study. Data were collected from 650 Lahu hill tribe women by simple random sampling. The interview forms with reliability coefficient and validity of 0.78 and 0.91 were administered to participants. The data were analyzed by using descriptive statistics and Chi-square test.

**Results:**

The 96.15% of the Lahu hill tribe women had taken Pap test and 74.31% obtained the screening more than once. The contraceptive use and frequency of Pap test obtaining significantly associated with cervical cancer screening (p<0.001). The participants showed good level of knowledge in cervical cancer and the screening test (64.31% and 76.46% respectively). Most of participants received the cervical cancer disease information (87.17%) and screening information (66.92%) from health care professionals, which may influence on well cervical screening co-operation.

**Conclusions:**

The suitable health promotion model should provide to promote knowledge, attitude and motivate continuous cooperation in cervical cancer screening among hill tribe women.

## Introduction

Cervical cancer is one of preventable and treatable forms of cancer. Human papillomavirus (HPV) vaccination, early detection, and early stage management are the effective cervical cancer prevention. Cervical cancer remains one of the serious diseases that threaten women’s lives. Globally, cervical cancer remains the third leading causes of cancer-associated mortalities among women (Bray et al., 2018; Sung et al., 2021). 

In Thailand, cervical cancer has also remained in the top three prevalence rate among female cancers for several years. Cervical cancer ranks are 2^nd^ most common female cancer and cause of cancer deaths in women aged 15 to 44 years. HPV information center reported that the estimate cervical cancer incidence rate for year 2018 is about 8,622 cases which is the 2nd leading cause of female cancer. There were about 5,015 deaths from cervical cancer which ranks as the 4th leading cause of female cancer deaths in Thailand (ICO/IARC Information Centre on HPV and Cancer, 2018). The number of cancer cases increased every year, whereas, cervical cancer screening rate decreased from 22.64 % in 2010 to 3.78 in 2014. There are three cervical cancer screening tests include cervical cytology (Pap smear or Pap test), primary HPV testing and cotesting (HPV testing in combination with cytology) (National Cancer Institute, 2020). The Pap smear is one of the tests that has been used for decade in a large population, and has been shown to effectively decrease mortality of cervical cancer (WHO, 2014; National Cancer Institute, 2020; Fontham et al, 2020). 

Thai government provides Universal Health Coverage (UHC) system to ensures an health care access equity of all citizens including healthcare services, health personnel, health information, medicines and medical instruments, good governance, and the health financing system (NHSO, 2015). The Ministry of Public Health requires that all Thai women in reproductive age have access to cervical cancer screening. Pap test is widely used to screen for cervical cancer in Thailand because of its simplicity and cost-effectiveness. The aim of cancer screening is to reduce death from the disease. When pre-cancer lesions are detected and remedied, the incidence of cancer cervix might decrease (Adeyemi et al., 2018; Abu and Arun, 2020). 

In Northern Thailand, the cervical cancer rate was 0.045 (Health Data Center, 2018) and various risk factors (such as early age at marriage (≤17 years), high frequency pregnancies and no/low education) were identified (Kritpetcharat et al., 2012; Wongwatcharakul et al., 2014). This area consists of the mountainous areas where a cervical cancer screening accessibility is limited and it may increase an incidence of deadly cervical cancer. Groups of people who live in a mountain area are identified as hill tribes such as Akha, Hmong, Karen, Hmong, Lahu, Yao, Mein and others. Hill tribes are ethnically distinct minority groups who have a unique culture and lifestyle, differing from most of the population in the cities. The difference in culture and lifestyle of hill tribes might not have been accounted for in the design of screening strategy and the rate of cervical screening in this group of population was low (Wongwatcharakul et al., 2014). Such low uptake cervical screening rate might have resulted from poor knowledge about cervical cancer and screening, poor economic condition and/or religious prohibition (Kritpetcharat et al., 2012). A previous study found that Akha hill tribe women were the major group that took the Pap test screening, whereas few in Karen, Hmong, Lahu, Yao and others did so (Kritpetcharat et al., 2012). The factors that positive associated with up take cervical cancer screening among Hmong hill tribe include level of education, animistic religious beliefs, number of pregnancy and perceived risk of disease and the screening (Wongwatcharakul et al., 2014). Chiang Rai is the province in Northern Thailand with unique geography and variety of ethnics. Lahu hill tribe is a main ethnic who live in mountain area with agriculture occupation. They have unique culture and lifestyle. Illness always believe attribute sough from supernatural cause. Ancestor soul-recall ceremony was performed to remedy the sickness. The health care services accessibility of Lahu hill tribe people in limit including Pap test. Last two years succeeded on cervical cancer screening (Pap test) in Lahu hill tribe women in one sub-district, Chiang Rai province was found. More than 75 percent of Lahu hill tribe women obtained the Pap-test screening each year (Health Data Center, 2018). The research project aimed to determine factors associated with success on Pap test screening among Lahu hill tribe women in order to develop a suitable health promotion plan for women in other tribes. 

## Materials and Methods

*The cervical cancer screening in study setting*

According to strategic plan to reduce health risk factors of the Ministry of Public Health, Thailand, the Pap test outreach service was provided for all Lahu hill tribe women every year by health care professional. The community leaders were contacted one month before screening service. All information for the Pap test screening was announced two weeks before the screening by health care professional, community leaders and village health volunteers. The collected sample was done by health care professional in private room at village meeting center. Any women who could not take the screening as schedule (such as during the period, during pregnancy), they could obtain the screening at health promotion hospital’s Pap test clinic every Thursday. 

*Study design*

A cross-sectional analytic survey was performed at a district in Chiang Rai, Thailand in January, 2018. The Lahu hill tribe communities in the district were the selected area in this study. It is located in remote area about 126 kilometers from the center of Chiang Rai. Two sub-districts were randomized to enroll in the study. There were 650 Lahu hill tribe women who convenient to provided information and met the inclusion criteria (age of 30 to 60 years old with more than one year of residence) were interviewed face-to-face lasted for 20 to 30 minutes. 

*Materials*

The interview forms were used to obtain information on the socio-demographic characteristics (ethnicity, age, highest education level, marital status, pregnancy history), the uptake of Pap test (ever, never, number of Pap test experiences), and the source of cervical cancer and screening knowledge. There were 20 yes-no questions about the knowledge of cervical cancer and 11 questions on cervical screening. The score of 1 was given for the correct answer and 0 for the incorrect one. The reliability and validity of interview form were 0.78 and 0.91. Translation of interview form into Lahu language and back in Thai language were performed by Lahu hill tribe volunteer who understand both Lahu and Thai language. 

This study has been approved by Mae Fah Luang University Research Ethics Committee on Human Research (reference No.109/2560) and permission from Mae Je dee Mai health promotion hospital director. Informed consent has been obtained from the participants.

*Statistical analysis*

Descriptive statistics were used to analyze the participants’ characteristics. The Chi-square test was performed to determine the factors associated with Pap test uptake. P values less than 0.05 were considered to be significant.

## Results

The most participant had experience on Pap test screening (96.15%) and 89.08% were married. The mean age of participant was 42.28 and received primary education (57.08%), only 4.77% had a bachelor’s degree. The main occupation was agricultural (70.31%). The mean ages of first marriage, first sexual intercourse and first birth were 17, 17 and 19 years old, respectively. The most employed fertility control was contraceptive injection (92.77%) (Table 1). The top three reasons for obtaining the Pap test include; interest in own health (94.88%), recommendation from healthcare professionals (35.68%) and recommendation from village health volunteers (28.32%). The perceived barriers were health misperception, fear and embarrassment (Table 2). 

*Knowledge about cervical cancer*

The cervical cancer knowledge score showed an average of 13.29 (2.55) out of a total score of 20. 

The top five highest proportion of answered correctly were AIDs and HIV infection Women increase risk of cervical cancer (79.08%), early onset of sexual activity increases risk of cervical cancer (78.46%), women had history of sexually transmitted infections increase risk of cervical cancer (77.85%), women do not obtain cervical cancer screening increase risk of cervical cancer (77.69%), and marriage women have high risk of cervical cancer (75.85%) (Table 3). Nearly half of participants believed that Ethnic women have low risk of cervical cancer (47.08%) and disrespect of ethnic culture and village tradition can cause cancer (53.54%). 

*Factors associated the Pap test obtaining*

The study found that fertility control and frequency of Pap test screening significantly associated with Pap test uptake in Lahu hill tribe women (P<0.001). In the contrary, education, perceived risk of cervical cancer and perceived useful of cervical screening were not significantly related to the Pap test obtaining. 

**Table 1:** Percentage Distribution of Participants According to Their Demographic and Obtained Pap-Test in Lahu Hill Tribe Women (n = 650)

Characteristics	n	%
Age at interview (years)		
30-40	294	45.23
41-50	259	39.85
51-60	97	14.92
	= 42.28 SD = 7.89	
Education		
No education	129	19.85
Primary school	371	57.08
High school	58	8.92
Vocational Certificate	61	9.38
Bachelor degree	31	4.77
Occupation		
Agricultural worker	457	70.31
Commercial	111	17.08
Labor	81	12.46
Government service	1	0.15
Married status		
Single	3	0.46
Married	579	89.08
Widow/ Divorce	68	10.46
Age at first marriage (n= 647)		
< 18 years	379	58.58
≥18 years	268	41.42
	= 17.44 SD = 3.89	
	Min = 10 Max= 39	
Age at first sexual intercourse (n= 647)		
< 18 years	394	60.62
≥18 years	253	38.92
	= 17.18 SD = 3.70	
	Min = 10 Max= 32	
Number of married		
No	3	0.46
1	457	70.31
2	135	20.77
≥3	55	8.46
Age at first pregnancy (n=630)		
< 20 years	350	55.56
≥20 years	280	44.44
	= 19.76 SD = 4.79	
	Min = 11 Max= 34	
History of abortion (n=630)		
No	523	83.02
1	102	16.19
≥ 2	5	0.79
Characteristics	n	%
Number of children		
No	20	3.08
1	94	14.46
2	269	41.38
3	178	27.38
≥4	89	13.7
Fertility control (Contraception)		
No	3	0.46
Oral contraceptive pill	32	4.92
Contraceptive injection	603	92.77
Surgical sterilization	12	1.85

**Table 2:** History of Pap Test and Perceived of Cervical Cancer and Cervical Screening (n =650

	n	%
The Experience of taking Pap test		
No	21	3.23
1	146	22.46
2	235	36.15
3	97	14.92
4	11	1.69
5	56	8.62
≥6	84	12.92
Reason for taking screening (n=625)*		
Want to know health status	593	94.88
Health care professional recommendation	223	35.68
Village health volunteer recommendation	177	28.32
Fear of illness	66	10.56
Relative /Cousin recommendation	26	4.16
Relative/Cousin/Friend had cervical cancer history	25	4
Reason for not taking screening (n=25)*		
I am healthy and have no risk factors	21	84
Fear to found the abnormal result	11	44
Shy	8	32
Cousin do not permission	6	24
No vehicle	5	20
No time	2	8
Perceived symptoms and risk of developing cervical cancer		
Poor (0-12)	232	35.69
Good (13-20)	418	64.31
Perceived the method and useful of Pap test		
Poor (0-6)	153	23.54
Good (7-11)	497	76.46
Received information about cervical cancer		
Never	3	0.46
Ever	647	99.54
Source of information about cervical cancer (n= 647)*		
Health care professional	564	87.17
Village health volunteer	297	45.9
Television, Radio	128	19.78
Women who had experience on Pap test	66	10.2
Community leader announce	5	0.77
Source of information about Pap test*		
Health care professional	435	66.92
Village health volunteer	219	33.69
Television, Radio	29	4.46

* The participants could choose more than one answer; therefore, the percentage was calculated by using the number of responses.

**Table 3:** Knowledge about Cervical Cancer (n=650)

	Respondent’s answer, n (%)	
	Correct	Incorrect
1. The watery or purulent vaginal discharge are early symptom of cervical cancer	396 (60.92%)	254 (39.08%)
2. Postcoital bleeding is early symptom of cervical cancer	363 (55.85%)	287 (44.15%)
3. Irregular or heavy vaginal bleeding (not menstruation) is early symptom of cervical cancer	401(61.69%)	249 (38.31%)
4. Irregular vaginal bleeding in menopause women is early symptom of cervical cancer	373 (57.38%)	277 (42.62%)
5. Treatment of early-stage cervical cancer can prevent the development of invasive cervical cancer and reduce mortality	453 (69.69%)	197 (30.31%)
6. pelvic or lower back pain are symptoms of Advanced cervical cancer	474 (72.92%)	176 (27.08%)
7. Ethnic women have low risk of cervical cancer *	344 (52.92%)	306 (47.08%)
8. Early onset of sexual activity (younger than 18 years old) increases risk of cervical cancer	510 (78.46%)	140 (21.54%)
9. Marriage women have high risk of cervical cancer	493 (75.85%)	157 (24.15%)
10. Women have multiple sexual partners increase risk of cervical cancer	451 (69.38%)	199 (30.62%)
11. Early age at first birth women (younger than 20 years old) increase risk of cervical cancer	483 (74.31%)	167 (25.69%)
12. Women give birth more than 3 times increase risk of cervical cancer	463 (71.23%)	187 (28.77%)
13. Prolong oral contraceptive use (more than 5 years) increase risk of cervical cancer	442 (68.00%)	208 (32.00%)
14. Women had history of sexually transmitted infections (eg, Chlamydia trachomatis, genital herpes) increase risk of cervical cancer	506 (77.85%)	144 (22.15%)
15. AIDs and HIV infection Women increase risk of cervical cancer	514 (79.08%)	136 (20.92%)
16. The frequent ferment food consumption increase risk of cervical cancer	314 (48.31%)	336 (51.69%)
17. Smoked women risk of cervical cancer	452 (69.54%)	198 (30.46%)
18. Work hard women risk of cervical cancer *	352 (54.15%)	298 (45.85%)
19. Cervical cancer is occur in women do not respect of ethnic culture and village tradition *	348 (53.54%)	302 (46.46%)
20. Women do not obtain cervical cancer screening increase risk of cervical cancer	505 (77.69%)	145 (22.31%)

*, Answer is false

**Table 4:** Factors Associated with Obtained Pap-Test in Lahu Hill Tribe Women (n=650)

	Screened (n=625)		No screen (n=25)		X^2^	P-value
	n	%	n	%		
Age at interview (years)						
30-40	284	45.44	10	40		
41-50	248	39.68	11	44	0.29	0.865
51-60	93	14.88	4	16		
Education						
No education	127	20.32	2	8	2.294	0.13
Education	498	79.68	23	92		
Age at first sexual intercourse (n= 647)						
< 18 years	378	60.77	16	64	0.105	0.746
≥18 years	244	39.23	9	36		
Age at first pregnancy (n=630)						
< 20 years	335	55.28	15	62.5	0.487	0.485
≥20 years	271	44.72	9	37.5		
History of abortion (n=630)						
No	501	82.67	22	91.67	1.324	0.25
Yes	105	17.33	2	8.33		
Fertility control (Contraceptive)						
No	1	0.16	2	8	32.162	<0.001*
Yes	624	99.84	23	92		
Perceived risk of developing cervical cancer						
Poor (0-13)	224	35.84	8	32	0.154	0.694
Good (14-23)	401	64.16	17	68	0.154	
Perceived the method and useful of Pap test						
Poor (0-6)	150	24	3	12	1.923	0.165
Good (7-11)	475	76	22	88		
Frequency of Pap test obtaining						
0 - 1 time	145	23.2	22	88	52.871	<0.001*
≥ 2 times	480	76.8	3	12		

* p<0.001

## Discussion

A cervical cancer is a significant public health problem in Thailand. Thai government provides a Pap test screening to identify a cervical cancer high risk woman via local primary health care. The high rate of Pap test screening was found among Lahu hill tribe women in one sub-district where locate in remote area of Chiang Rai province. 

*Interpersonal factors influencing Pap test screening*

Statistically significant correlation between the frequency of Pap test screening and cervical cancer screening was observed in this study. This study also found that most screened women had a Pap test experience more than once which is according to previous study in Vietnam. It was found that the Vietnamese women who had Pap test experience significantly intended to take a Pap test in the next time (P < 0.013) (Lee et al., 2016). The Fertility control showed significantly influenced on Pap test obtaining. The result related to previous studies which revealed that contraceptive use was associated the Pap test obtaining (Vinekar et al., 2015; Chosamata et al., 2015) while Rashied and Abbas’s study (2014) was found that previous abortion was one barrier factor in taking the Pap test due to the perception of Pap test being painful. The results support awareness on their health status that might be influencing on well cervical screening co-operation. The health concern of Lahu hill tribe women might be from their culture on women role. Their role was not only giving birth but women did also the labor work same as men (Ma, 2013). Lahu hill tribe women believed that woman is worthless person if do nothing and do not give birth after married. They must be healthy to be able to take care of their family matters. Therefore, women would take care of health status for their role. Other risk factors (i.e. age at first marriage, age at first intercourse, age at first pregnancy, occupational and cervical cancer knowledge) were not related to screening attendance of Lahu hill tribe women. This result differs from a previous study among hill tribes which found that the factors positively associated with Pap test attendance were the number of pregnancies, received information about cervical cancer and perceived risks of cervical cancer (Wongwatcharakul et al., 2014). 

*Social factors influencing Pap test screening*

Formal education might be no effect on perceive cervical cancer knowledge in Lahu hill tribe women while healthcare professionals and community health volunteers were the most important agents that provided knowledge about cervical cancer and screening. This information implies the trust and perceive of Lahu hill tribe people in health care professionals. They provided the outreach health service about four times per year and once onsite Pap test service a year. Outreach screening service is a primary care unit that improved access to healthcare service for Lahu hill tribes. Good healthcare service and information from healthcare professionals and village health volunteers might have a positive effect on Lahu hill tribe people’s trust, their health interest, and the intention to participate in healthcare service, especially Pap test. According to previous study (Lynge et al., 2012), the keys to successful screening program are societal acceptance, local ownership, and effective coordination along with the best evidence-base practice. The sustained community outreach is necessary to increase awareness of cancer screening recommendations, identify high-risk individuals, and suggestion the health resources (William et al., 2018). Lahu village health volunteers also the important person who publicize the information about cervical cancer and screening (Table 2). The village health volunteers are those chosen by their community members not only for being public-minded, generous, and willing to help those in health need, but also comparatively knowledgeable and concerned about health matters (Chuengsatiansup and Suksuth, 2007). They could communicate both in their language and Thai, so that they helped to translate Lahu language to Thai language and vice versa when necessary. Most of them had worked as a village health volunteer for more than five years. Those reasons might explain why all volunteers were accepted by the community and played important role in village healthcare. Therefore, good participation from those groups of people is a very important factor that could lead to Pap test uptake among Lahu hill tribe women. The results from this study are related to many studies, revealing that the facilitating factors to cervical cancer screening include access to information and transportation, trust in healthcare professionals, free service, and reminder by telephone call (Abdullahi et al., 2009; Arabaci and Ozsoy, 2012; Bellinger, 2015; Ashtarian et al., 2017) while the barriers were the lack of knowledge, fear, shyness, embarrassment, and past negative experience (Arabaci and Ozsoy, 2012; Ersin and Bahar, 2013; Daley et al., 2013) and the perception of being healthy (Rashied and Abbas, 2014). The observation during collect data found that the Lahu hill tribe societies have good relationship overall. Senior women share their knowledge and experience on screening, as well as teaching and motivating new generations to obtain the screening. Their homes are close to one another without any fences. Closed relationships among Lahu hill tribe people increase the chance of spreading community news and information on Pap test taking to the members. Their unity is probably one of the keys to successful screening. 

*Risk factors on cervical cancer*

It is noted that most participants showed the risk factors associated with cervical cancer, including early onset of sexual activity with the mean age of about 17 years old, early pregnancy and early age at first birth with the mean of 19 years old (Table 1). Previous study revealed that the significantly increase of cervical cancer risk in women whose age at early intercourse debut age 18 -20 years old (Xiao et al., 2018). Higher pregnancy rate and number of live births were also found in this study (Table 1). Those factors significantly associated with increased risk of cervical cancer (Xiao et al., 2018). The results correspond to the previous study which found that hill tribe women had several risk factors of cervical cancer, including early age at first sexual intercourse, marriage, and pregnancy, the number of pregnancies, and delivery (Kritpetcharat et al., 2012; Wongwatcharakul et al., 2014). However, there is a very low incidence rate of cervical cancer in Lahu hill tribe women even though they demonstrated high risk factors. Last year’s cervical cancer incidence rate is zero percent, which is lower than the incidence rate in Chiang Rai province at age 40-49, 50-59 and >60 years old with the percentage of 0.04, 0.07 and 0.10 respectively and the nationwide rate in the age 15-39, 40-49, 50-59 and >60 years old with the percentage of 0.01, 0.05, 0.09 and 0.09 respectively (Health Data Center, 2018) . 

The goal of Thai universal health coverage policy is equality to access to healthcare services for all Thai citizens. Therefore, good coordination between healthcare professionals and key persons in the community (health volunteers and community leaders) is a crucial factor that could lead to the uptake of Pap test among Lahu hill tribe women. The universal health coverage policy of Thai government should be aware of the capability and transcultural factors to be able to provide good quality health service for all hill tribe population. 

*Limitation and Implication*

The difficulty in communication and transportation are the main limitations of this study. Most participants could not communicate with the researchers so we had to use the translator for the questionnaire. This could have affected the full understanding of the questions. As for area accessibility, some areas were hard to reach such as in the higher area which could not be accessed by cars, and some of which were dangerous private areas.

The results from the study could serve as a reference for healthcare professionals in empowering Lahu hill tribe women to take care of their health. Lahu hill tribe women can be the role model to share their experience in taking the Pap test to other women who are still hesitant to obtain the screening. The outreach screening services and publicized the cervical cancer protection may also induce the good cooperation. These strategies might increase and maintain the success rate of cervical cancer screening among others hill tribe and indigenous women.

In conclude, this study found that the concern on health status of Lahu hill tribe women was the important factor to obtained Pap test screening. Health care professionals was the key person who publicized the cervical cancer and screening information, which may influence the successful of screening. The suitable health promotion model should provide to promote knowledge, attitude and motivate continuous cooperation in cervical cancer screening among hill tribe women. Furthermore, the hill tribe cultures and participation from health volunteers should be concerned as well. 

## Author Contribution Statement

SI developed research concept, funding management, collected data, interpreted the analysis, wrote the main manuscript, prepared Tables 2, 4 and reviewed the manuscript. KN analyzed data, prepared Tables 1, 3 and reviewed the manuscript. PK collected data and reviewed the manuscript.

## Data Availability

The raw data available upon reasonable request from the corresponding author.
